# Statistical Evaluations of Variations in Dairy Cows’ Milk Yields as a Precursor of Earthquakes

**DOI:** 10.3390/ani7030019

**Published:** 2017-03-08

**Authors:** Hiroyuki Yamauchi, Masashi Hayakawa, Tomokazu Asano, Nobuyo Ohtani, Mitsuaki Ohta

**Affiliations:** 1 Department of Animal Science and Biotechnology, Azabu University Graduate School of Veterinary Science, 1-17-71 Fuchinobe, Chuo-ku, Sagamihara, Kanagawa 252-5201, Japan; ohtani@azabu-u.ac.jp; 2 Hayakawa Institute of Seismo Electromagnetics Co. Ltd., UEC (University of Electro-Communications) Incubation Center, 1-5-1 Chofugaoka, Chofu, Tokyo 182-8585, Japan; hayakawa@hi-seismo-em.jp (M.H.); tomokei0929@gmail.com (T.A.); 3 Department of Human and Animal-Plant Relationships, Tokyo University of Agriculture, 1737 Funako, Atsugi, Kanagawa 243-0034, Japan; mo205684@nodai.ac.jp

**Keywords:** dairy cows, earthquake precursors, unusual animal behavior, milk yields, subionospheric very low frequency/low frequency (VLF/LF) propagation

## Abstract

**Simple Summary:**

There are many reports of abnormal changes occurring in various natural systems prior to earthquakes. Unusual animal behavior is one of these abnormalities; however, there are few objective indicators and to date, reliability has remained uncertain. We found that milk yields of dairy cows decreased prior to an earthquake in our previous case study. In this study, we examined the reliability of decreases in milk yields as a precursor for earthquakes using long-term observation data. In the results, milk yields decreased approximately three weeks before earthquakes. We have come to the conclusion that dairy cow milk yields have applicability as an objectively observable unusual animal behavior prior to earthquakes, and dairy cows respond to some physical or chemical precursors of earthquakes.

**Abstract:**

Previous studies have provided quantitative data regarding unusual animal behavior prior to earthquakes; however, few studies include long-term, observational data. Our previous study revealed that the milk yields of dairy cows decreased prior to an extremely large earthquake. To clarify whether the milk yields decrease prior to earthquakes, we examined the relationship between earthquakes of various magnitudes and daily milk yields. The observation period was one year. In the results, cross-correlation analyses revealed a significant negative correlation between earthquake occurrence and milk yields approximately three weeks beforehand. Approximately a week and a half beforehand, a positive correlation was revealed, and the correlation gradually receded to zero as the day of the earthquake approached. Future studies that use data from a longer observation period are needed because this study only considered ten earthquakes and therefore does not have strong statistical power. Additionally, we compared the milk yields with the subionospheric very low frequency/low frequency (VLF/LF) propagation data indicating ionospheric perturbations. The results showed that anomalies of VLF/LF propagation data emerged prior to all of the earthquakes following decreases in milk yields; the milk yields decreased earlier than propagation anomalies. We mention how ultralow frequency magnetic fields are a stimulus that could reduce milk yields. This study suggests that dairy cow milk yields decrease prior to earthquakes, and that they might respond to stimuli emerging earlier than ionospheric perturbations.

## 1. Introduction

There have been numerous studies on precursors of earthquakes [1,2,3,4,5,6,7,8,9,10,11]. These studies have mainly focused on pre-seismic unusual physical and/or chemical variations near the epicenters, such as electromagnetic emissions, ionospheric perturbations, radiation belt electron precipitation and radon gasses, that emerge prior to earthquakes [1,2,3,4,5,6,7,8,9,10,11]. In particular, it has recently been reported that some precursors such as electromagnetic and radon anomalies showed statistical correlations with earthquakes [12,13,14,15,16]. Additionally, there have been many reports of unusual animal behavior (UAB) prior to earthquakes. UABs accounted for approximately half of all reports of macroscopic anomalies identified in posteriori surveys, which also included abnormal sounds, earthquake lights, earthquake clouds, ground deformation, and abnormalities in the ground water [17,18,19]. However, most reports on UAB are based on qualitative rather than quantitative observations. As an example of quantitative UAB, changes in the locomotive activities of mice before large earthquakes were reported by Yokoi et al. [20] and Li et al. [21]. Grant et al. [22] recently revealed, by the use of motion-triggered cameras, that wild animal activity in various species declined prior to the Contamana earthquake, with a magnitude (M) of 7.0. However, these reports were case studies for single large earthquakes. Few studies have found statistical correlations between earthquakes and UAB using longitudinal quantitative observations.

UAB prior to earthquakes includes stress or emotional responses to physical or chemical anomalies, although the mechanism by which these anomalies are sensed remains unknown. We hypothesize that the milk yields of dairy cows, while not a behavior, might be useful as an earthquake precursor because milk yields are decreased by various stressors [23,24,25]. Specifically, Regalma et al. [25] reported that cows exhibited responses including decreases in milk yields in response to small stray voltage in the ground, and Rushen et al. [23,24] reported decreases following handling by unfamiliar people, moves to novel places and social isolation. In addition, milk yields are measured every day by farmers as a normal part of their work. There are also reports that cows exhibited UABs prior to earthquakes [26,27]. Prior evidence in support of this hypothesis comes from a study that reported milk yields from cows located 340 km from the epicenter decreased from three to six days before the 2011 earthquake off the Pacific coast of Tohoku in Japan (Tohoku earthquake; Mw 9.0) [28]. However, this was a case study of one extremely large earthquake. To assess whether milk yields decrease prior to earthquakes, a time-series analysis of the correlation between milk yields and earthquakes with various magnitudes (Ms) using long term data is necessary. To confirm the value of a certain phenomenon as an earthquake precursor, it is not only necessary to evaluate by the time-series analysis but also to estimate the performance level using retrospective earthquake prediction. Mathematical methodologies and parameters to evaluate the performance of predictors have been described in previous studies [29,30].

An electromagnetic anomaly in the ionosphere prior to earthquakes is one well-known precursor. Very low frequency/low frequency (VLF/LF) subionospheric propagation data have recently been used to monitor lower ionospheric perturbations associated with earthquakes. Statistical correlations with earthquakes have been confirmed in previous studies [13,14], and there are some theoretical mechanisms regarding this anomaly [31,32]. Interestingly, on the same day the milk yields decreased [28], anomalies in the VLF/LF propagation signal emerged [33] in studies on the Tohoku earthquake. A comparison of the milk yield anomalies and other well-known precursors could help elucidate mechanisms associated with UAB prior to earthquakes.

The aims of this study are to elucidate whether the milk yields of dairy cows decreased before various earthquakes and to confirm whether precursory decreases in milk yields are an explainable phenomenon from a scientific point of view by comparison with VLF/LF propagation data. To achieve our aims, we estimated the relationships between milk yields and earthquakes by time-series analyses and preliminarily estimated the performance level using milk yields, with some definitions of anomalies, and earthquakes with varying thresholds regarding distance from epicenters and M.

## 2. Materials and Methods

### 2.1. Milk Yields

**Figure 1:**
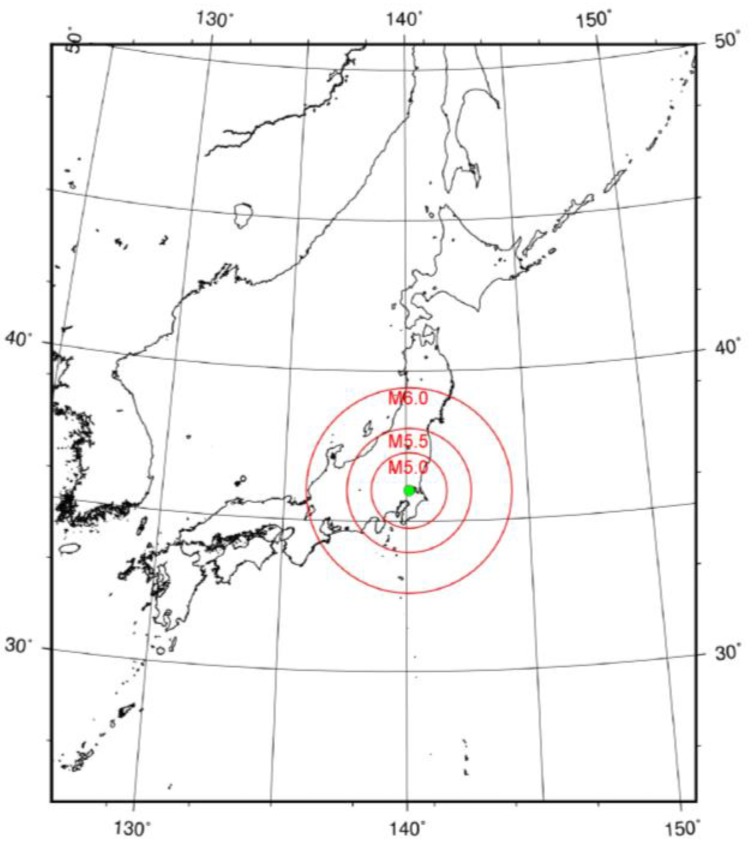
The radius (km) calculated according to the Dobrovolsky radius condition with magnitudes (Ms) = 5.0, 5.5 and 6.0. The red circles represent the maximum range for earthquakes of each M. The solid green circle represents the location of the farm used to observe milk yields.

**Figure 2:**
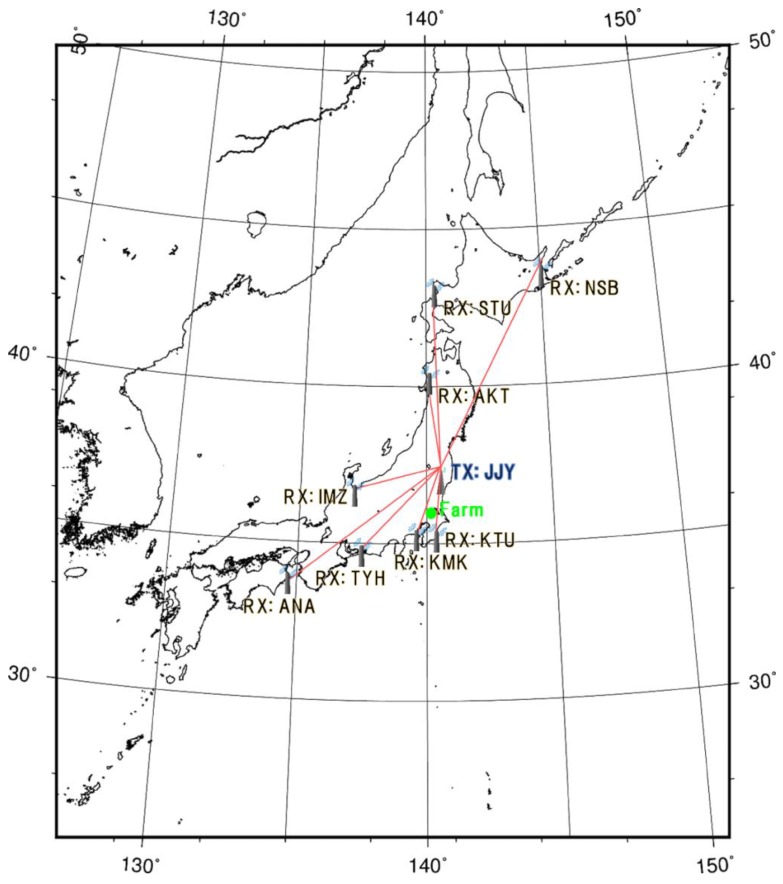
The location of the farm used to observe milk yields, eight observatories and the Japanese low frequency transmitting station (JJY) for very low frequency/low frequency (VLF/LF) propagation data. Red lines represent the propagation path from JJY to the eight observatories. NSB = Nakashibetsu; AKT = Akita; IMZ = Imizu; KTU = Katsuura; KMK = Kamakura; TYH = Toyohashi ANA = Anan; STU = Suttu.

With the aid of a farm in Ibaraki prefecture of Japan, we analyzed the daily milk yields of 48 Holstein cows from 1 January 2014 to 31 December 2014. The location of the farm is shown in Figure 1 and Figure 2. The milking process was as follows; the cows were brought into the milking parlor and then milked by machines. They were individually identified by tags, and milk yields were assessed using electronic milk meters. The milking frequency was twice a day. The data measured were transferred to computers, and we used the total milk yield per cow per day. Variability in milk yield due to some physiological and environmental effects were corrected for before statistical analyses, based on our previous work [28]. Milk yields from dairy cows increase for approximately four to eight weeks postpartum and gradually decrease thereafter, with lactation being complete by approximately forty weeks. First, we removed the effect of the number of days after calving using Wood’s lactation curve model [34]. Individual milk yields during one lactation period were estimated by the use of Wood’s model, as follows:

(1) $$y_{t}\;=\;a\times n_{t}^{b}\times e^{-c\times n_{t}},$$

where y_t_ is the expected milk yield in time t, n_t_ is the number of days after calving, e is natural logarithm, and a, b and c are parameters. These parameters were estimated using a general linear model (glm function in R; version 3.3.0, The R Foundation for Statistical Computing, Vienna, Austria) after a logarithmic transformation [35,36]. Table 1 shows estimated parameters for individual lactation curves. Then, residual values I of milk yields (dM-I) were calculated by subtracting these predicted values from the actual measurement values of the current day:

dM-I_t_ = M_t_ − pM-I_t_, (2)

where M_t_ is the milk yield for a current day and pM-I_t_ is the predicted value for the current day estimated by Wood’s model.

Factors affecting milk yields in dairy cows include ambient temperature and humidity as well as the number of days after calving. The temperature–humidity index (THI) is often used as an index of heat stress for cows. West et al. [37] reported that milk yields begin to decrease when the THI exceeds 72. These effects from environmental conditions stem from decreases in food consumption due to heat stress. Therefore, increased temperature might affect milk yields after several days rather than on the current day. West et al. reported that THI during hot periods had the greatest impact on milk yields two days later [37]. This effect on heat stress should be removed to elucidate the relevance to earthquakes. Second, we calculated the daily THI values during the observation period using mean temperature and mean relative humidity from Japan Meteorological Agency’s meteorological observatory closest to the farm. The formula to calculate the THI is

THI_t_ = (1.8 T_t_ + 32) − (5.5 + 0.055 H_t_) × (1.8 T_t_ − 26), (3)

where T is the dry bulb temperature in °C and H is the relative humidity in % [38]. The critical point in THI at which milk yields start to decrease and the linear relationship between THI (above the critical point) and milk yields were estimated using a two-phased regression model [39]. The model formula is:

(4) $$\begin{array}{cc} y_{t}=a+bx_{t-2}, & \left(x\geq x0\right)\\ y_{t}=c, & \left(x<x0\right) \end{array}$$

where y_t_ is the mean dM-I at time t, x_t−2_ is the THI at the relevant lag, x0 is the critical point in THI, and a, b and c are parameters. The estimated model was used to calculate the expected daily milk yields from the THI values. Table 2 shows estimated parameters and the critical point. The residual values of the milk yields (dM-II) were calculated by subtracting these predicted values from dM-I:

dM-II_t_ = dM-I_t_ − pM-II_t_, (5)

where dM-I_t_ is the milk yield after removing the effect of calving time and pM-II_t_ is the predicted value based on the THI values. The dM-II data might have trend variation, which is inappropriate for time-series analyses. Therefore, final variations in milk yields were calculated by the partly-changed equation described by Maekawa et al. [14] and Hayakawa et al. [33]. That is, we calculated residual values in the milk yields as:

dM-III_t_ = dM-II_t_ − <dM-II_t_>, (6)

where dM-II_t_ is the milk yield after removal of two effects at time t and <dM-II_t_> is the 7-day backward moving average at the same time t. These final variations (dM-III) were used in analyses regarding the relevance of the data to earthquakes.

### 2.2. Cross-Correlation Analyses

To examine statistical correlations between changes in milk yields and earthquakes, a cross-correlation analysis was applied. The information on occurrence date, location, M, and depth of earthquakes were obtained from the Japan Meteorological Agency. To examine the relationships between milk yields and earthquakes, it was necessary to select thresholds regarding the distance from the epicenter. We used earthquakes that satisfied the Dobrovolsky radius condition (DRC). According to the DRC, the effective precursory manifestation zone depends on the M of earthquakes and can be calculated as:

r (km) = 10^0.43M^, (7)

where r is the radius from the epicenter and M is the magnitude [40]. Figure 1 shows the Dobrovolsky radius for some Ms.

The presence or absence of the occurrence of earthquakes on a given day was treated as a binary outcome (0/1). It was necessary to count multiple earthquakes on a given day as a single occurrence, because the milk yield data were collected only once per day. In a previous study, Maekawa et al. [14] used effective magnitude (Meff), which was calculated by integrating the released energy of earthquakes that occurred within one day and converting it back into M. The released energy was calculated using the Richter scale [41], as follows:

E = 10^(4.8+1.5M)^, (8)

where E is the released energy and M is the magnitude.

We also calculated this Meff from earthquakes with M > 2.0 as the active level of earthquakes. Then, we used dates with Meff > 5.0, 5.5 and 6.0 in the analyses. The analyses were performed using the ccf function in R (version 3.3.0). The 95% confidence intervals were set to 1.96/√(n − |k|).

### 2.3. Performance Evaluations of Binary Earthquake Forecasts

We also used a binary earthquake forecasting approach to evaluate the reliability of decreases in milk yields as a precursor for earthquakes. We obtained cross-tabulation tables consisting of the presence or absence of earthquakes and alarm or no alarm days from anomalies in milk yields (Table 3). To make these tables, it was necessary to determine the critical point that defines milk yields as being anomalous. In previous studies, values farther from the mean than twice the standard deviation (σ) were defined as anomalies [42]. We defined milk yields more than 1.5σ below the mean as anomalies to examine the relationship between relatively small decreases in milk yields and earthquakes. If milk yields were at abnormally low levels for more than two consecutive days, the dates were summarized as one anomalous occurrence because decreased milk yields were followed for a few days in a previous study [28]. To evaluate the continuity of anomalies in milk yields, we set two criteria regarding the definition of anomalies. In one criterion, we defined an anomaly as a decrease of more than 1.5σ for one day; in the other criterion, an episode was defined as an anomaly when the decreases continued over two days. ‘Alarm days’ were defined by the lag with the lowest cross-correlation coefficient. Assuming the temporal relationship between the presence of precursors and the occurrence of earthquakes can vary by a few days, the ‘alarm period’ included a margin of ± 4 days. To make the cross-tabulation tables, it was also necessary to determine the thresholds for M and the distance from the epicenters of earthquakes. We set three criteria on M, (i.e., >5.0, 5.5 or 6.0) because whether earthquakes with lower values of M led to decreases in milk yields was unclear. In addition, to confirm the appropriateness of the DRC as the sensitive area for milk yields, we considered two criteria regarding the distance from epicenters for targeted earthquakes. In one criterion, we defined an earthquake as relevant when it occurred within the DRC; in the other criterion, we defined an earthquake as relevant when it occurred within the DRC + 250 km. Finally, we made twelve cross-tabulation tables based on the varying criteria for the duration of anomalous milk yields and the targeted earthquakes. The details for each criterion are shown in Table 4.

These tables were used to calculate three scores indicating their performance level for binary earthquake forecasting.

Following Holliday et al. [30], we used hit rate (H), defined as

H = a/(a + b).(9)

Probability gain (PG), the ratio of the probability of an earthquake occurring in an alarm period divided by the probability of an earthquake occurring on any given day, is also an important parameter [29]. The formula for PG is:

PG = [a/(a + c)]/[(a + b)/(a + b + c + d)],(10)

Additionally, we calculated the successful rate of earthquake prediction (SEP) using the following equation:

SEP = a/(a + c),(11)

### 2.4. Comparison with Anomalies of VLF/LF Propagation Data

We collected subionospheric VLF/LF propagation data with the aid of the Earthquake Analysis Laboratory in Japan. This network for VLF/LF propagation has been in place since 2001 and revealed that anomalous propagations emerge prior to earthquakes [13,43]. In this study, we used eight observatories to confirm there were physical anomalies before relevant earthquakes: (1) Nakashibetsu (NSB); (2) Suttu (STU); (3) Akita (AKT); (4) Imizu (IMZ); (5) Katsuura (KTU); (6) Kamakura (KMK); (7) Toyohashi (TYH); and (8) Anan (ANA). We used the received signal from each observatory in the Japanese low frequency transmitting station (JJY; in Fukushima, 40 kHz). The locations of each observatory are shown in Figure 2, which also illustrates the propagation path from JJY to each observatory. To detect anomalies in VLF/LF propagation data, we analyzed the obtained propagation data in accordance with previous studies [44]. That is, we used daily average amplitude at nighttime and calculated residual values as dA(t) = A(t) − <A(t)>, where A(t) is the amplitude at time t on the current day, and <A(t)> is the running average at the same time t over ±15 days (i.e., 15 days before and after the relevant day). In parallel with milk yields, we defined the anomalies in VLF/LF propagation data by the occurrence of values that were decreased by more than 1.5σ.

## 3. Results

### 3.1. Cross-Correlation Analyses

**Figure 3:**
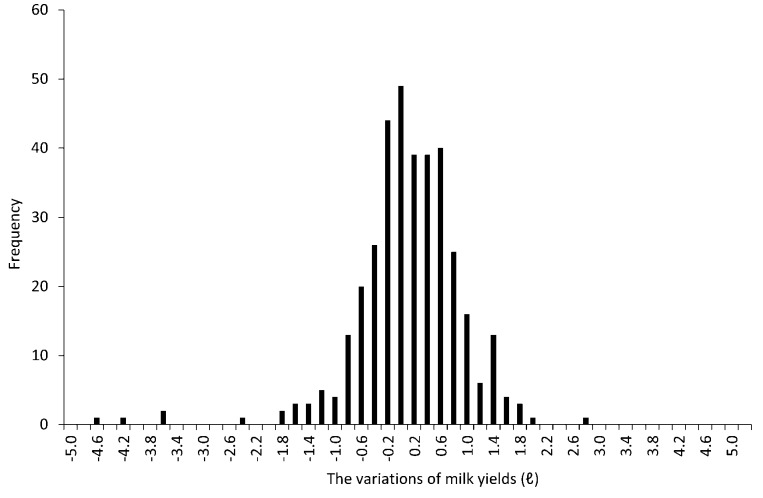
The distribution of the variations of milk yields.

**Figure 4:**
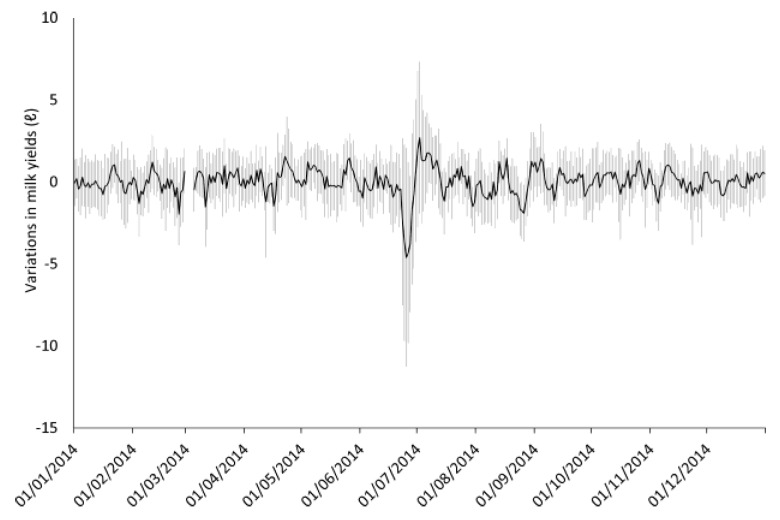
The variations of milk yields in the total observation period (Mean ± Standard deviation). The blank area indicates no observation.

**Figure 5:**
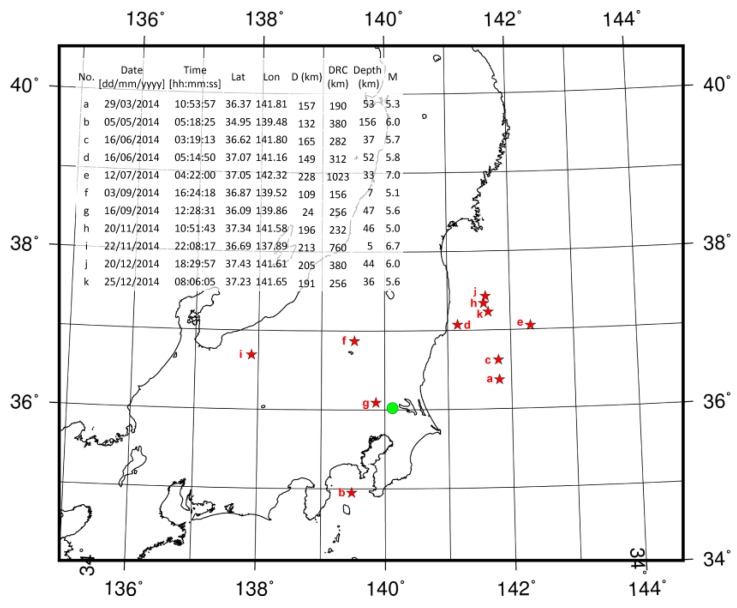
The detail of earthquakes which occurred during the days exceeding effective magnitude (Meff) 5.0. The red stars represent the locations of earthquakes. The green circle represents the location of the farm used to observe milk yields. The upper table shows the detail of earthquakes. The alphabets in the map correspond with those in the table. Lat = Latitude; Lon = Longitude; D = Distance from epicenters; DRC = Dobrovolsky radius condition.

**Figure 6:**
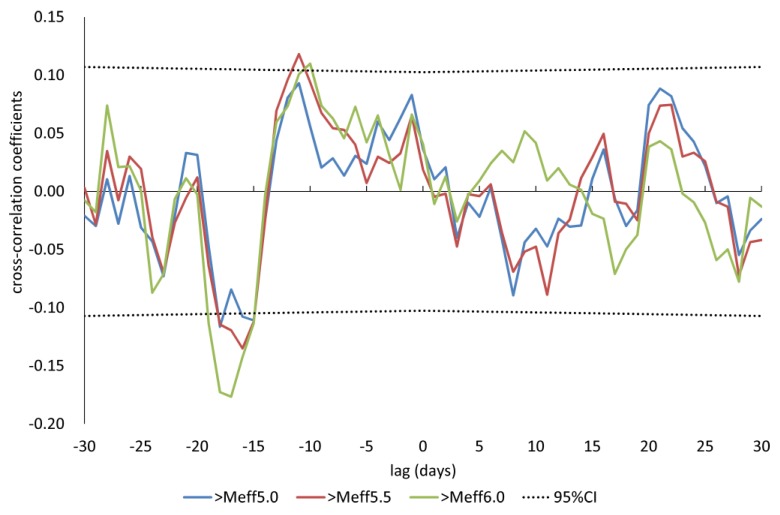
The results of cross-correlation analyses between milk yields and the dates exceeding each Meff; CI = confidence interval.

The number of lactating cows per day ranged from 18 to 36 (mean ± Standard deviation = 25.3 ± 4.2). The data on milk yields used in the analyses (mean ± S.D. = 0.016 ± 0.812) followed a normal distribution (one-sample Kolmogorov–Smirnov test, *p* = 0.47, ks.test function in R version 3.3.0) and exhibited stationarity (Phillips-Perron Unit Root Test, *p* < 0.01, p.p test function of tseries package in R version 3.3.0). Figure 3 shows the distribution of the milk yields. Figure 4 shows the mean variations of milk yields in the total observation period (361 days). Meff exceeded 5.0 on ten days, 5.5 on seven days and 6.0 on four days. The detail of earthquakes which occurred during the days exceeding Meff 5.0 is shown in Figure 5. Figure 6 shows the results of cross-correlation analyses between milk yields and each earthquake activity level. Significant negative coefficients were revealed approximately three weeks before the days exceeding each earthquake activity level, and the significances became clear for Meff > 5.5. Following this period, we found positive correlation coefficients; the coefficients 11 days before Meff 5.5 and 10 days before Meff 6.0 were statistically significantly positive. The positive coefficients continued until the days on which earthquakes occurred, although no other days’ coefficients were significant.

### 3.2. Binary Earthquake Forecasts

**Figure 7:**
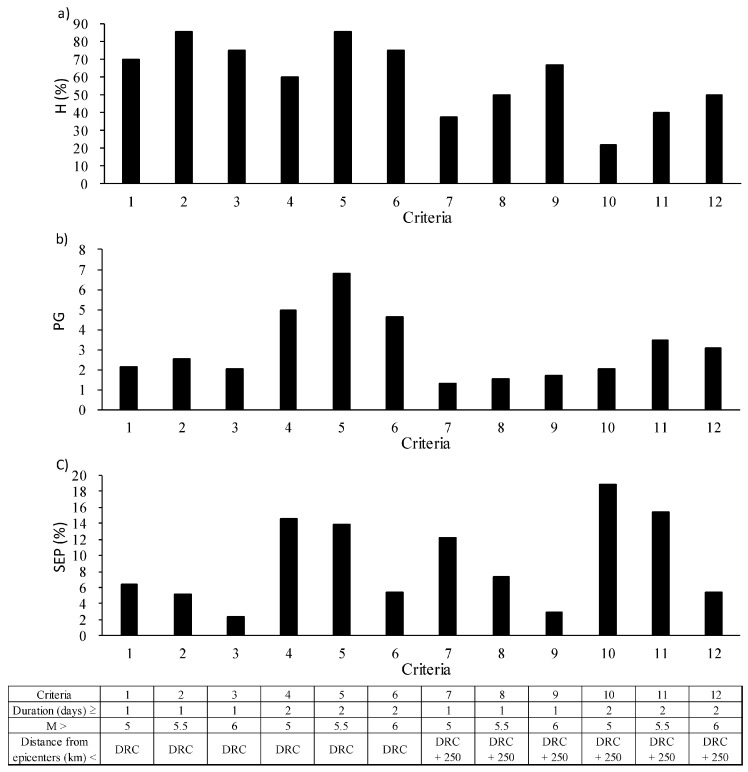
The scores used to estimate the cross-tabulation tables using the twelve criteria. (**a**) shows hit rate (H, %); (**b**) shows probability gain (PG) and (**c**) shows the successful rate of earthquake prediction (SEP, %). The lower table shows the details of the criteria.

On 16 June 2014, two earthquakes of similar M occurred in nearby regions (see Figure 5). We treated these earthquakes as one event with M greater than 5.5 that satisfied the DRC to prevent over- or under-estimation. On 31 days, milk yields decreased below the mean by more than 1.5σ. In 17 instances, the decrease persisted for more than two consecutive days. Table 5 shows cross-tabulation tables using the twelve aforementioned criteria. The scores of H, PG and SEP from the cross-tabulation tables are shown in Figure 7. Criteria 2 and 5 had highest H (85.7%). With respect to PG, criterion 5 had the highest score (6.8). The SEP for criteria 5 was 14.0%, which was not the highest value.

### 3.3. Comparison with Anomalies in VLF/LF Propagation Data

Based on the results of the binary earthquake forecasts, we compared milk yields with VLF/LF propagation data prior to earthquakes with M > 5.5 that satisfied the DRC and had high scores in both H and PG. Milk yields were defined as anomalous when values more than 1.5σ below the mean continued for more than two days. Table 6 shows the correspondence between the anomalies in milk yields, targeted earthquakes, and anomalies in VLF/LF propagation data. The anomalies in VLF/LF propagation data were presented in this table only if targeted earthquakes followed, because the focus of this study is milk yield in dairy cows. The results suggest that the anomalies in VLF/LF propagation data emerged prior to all of the earthquakes that accompanied anomalies in milk yields. However, in all cases, the lag times between anomalies in milk yields and earthquake occurrence were longer than those associated with anomalies in VLF/LF propagation. The lag times for milk yields and VLF/LF propagation were 17.7 and 10.5 days on average, respectively.

## 4. Discussion

### 4.1. Cross-Correlation Analyses

These analyses showed that milk yields decreased approximately three weeks before earthquakes. This result differed from the result reported regarding the leading time of decreases prior to the Tohoku earthquake of three to six days [28]. In our observation period, there were no earthquakes occurring near the hypocenter of the Tohoku earthquake; the earthquake nearest the epicenter of the Tohoku earthquake was 150 km away. This difference could be due to physical or chemical differences such as geostructural features in the epicentral zones. In addition to epicentral zones, two other differences stand out between these two studies. The M of the Tohoku earthquake was 9.0 while the M of targeted earthquakes in this study was a maximum of 7.0. The Tohoku earthquake also had many foreshocks starting two days before the main shock. Therefore, there are limitations regarding the comparability of the results in this study with those from the case study of the Tohoku earthquake. The significant cross-correlation coefficients became larger as Meff became larger. This indicates that the probability of decreasing milk yields increased, or the degree of decrease increased as the earthquake activity level increased. Reports have also suggested that anomalies in LF propagation signals before earthquakes increase with Meff or M [14,45]. However, several statistical studies suggest that anomalies in VLF/LF propagation signal emerge about one week before earthquakes [33]. This study suggests that the stimuli causing decreases in milk yields might occur before ionospheric anomalies. The increase in the cross-correlation coefficients appeared approximately 11 days before earthquakes. We subtracted the 7-day backward moving average from residuals in milk yields to remove trend variations in Equation 6. Therefore, the increase of the coefficients around −11 days likely indicates that actual milk yields did not increase but recovered. However, the positive coefficients lasted for approximately two weeks (i.e., until the day of the earthquake’s occurrence), although they did not exceed the significance threshold after day −10. The increased variability in milk yields seemed to gradually decline as the day of the earthquake approached. In addition to the pronounced decreases in milk yields, our study suggests there might also be an increase prior to earthquakes. Adverse effects prior to earthquakes have also been found in a study on the correlation between mental health and earthquakes [46].

### 4.2. Binary Earthquake Forecasts

Criterion 5 (i.e., in which anomalies included dates when milk yields were reduced by more than 1.5σ for more than two consecutive days and earthquakes with M > 5.5 that satisfied the DRC were included) had the highest H and PG scores. On the other hand, the highest SEP occurred in criterion 10, in which anomalies were defined as the dates when milk yields were reduced by at least 1.5σ for more than two consecutive days and targeted earthquakes were those with M > 5.0 and that satisfied the DRC + 250 km criterion. However, PG is obtained by dividing the SEP by the probability of an earthquake occurring, and earthquakes included in criterion 5, which included only earthquakes with M > 5.5 and those that satisfied the DRC, were stronger than those included in criterion 10 (i.e., M > 5.0 and DRC + 250 km). Therefore, it is likely that increasing the number of earthquakes by lowering the threshold of M and expanding the threshold distance led to an increase in accidental successful earthquake predictions. PG is approximately one when earthquakes and anomalies are uncorrelated. In this study, the PG of criterion 5 was 6.8. This value suggests that decreases in milk yields are related to subsequent earthquakes, in agreement with the result of the cross-correlation analyses. The aim of this study is to examine the reliability of decreases in daily cows’ milk yields as a precursor of earthquakes. Binary earthquake forecasts were used to evaluate the performance of this putative precursor. To identify the optimal precursor, we shifted the thresholds of several parameters regarding earthquakes and milk yields; however, the parameters considered were discrete and there were wide gaps between them. Therefore, to accurately understand the relationship between decreases in milk yields and subsequent earthquakes, it will be necessary to consider threshold values at smaller intervals. The SEP of criterion 5 was 14.0%, which is not high. The alarm period defined in these analyses was nine days per anomaly. If targeted earthquakes occurred during the alarm period, the remainder of the nine days were no longer considered alarm days (i.e., they were included in “d” in Table 3). Alarm days before the earthquakes were included in “c” in Table 3 (i.e., they were considered as days on which there was an alarm but no earthquake). Therefore, the unsuccessful rate of 86.0% consisted of not only alarm periods that were unrelated to earthquakes but also alarm days just prior to earthquakes.

### 4.3. Comparison with Anomalies of VLF/LF Propagation Data

In our observation period, anomalies in VLF/LF propagation data from regions that satisfied the DRC were observed prior to all earthquakes with M > 5.5. In previous studies, we found that the lag time of between decreased milk yield and the Tohoku earthquake [28] was similar to those of VLF/LF propagation anomalies [33]. However, in this study, the milk yields decreased approximately one week before VLF/LF propagation anomalies in all earthquakes that followed decreases in milk yields. These differences in the lag times were supported by the results of the cross-correlation analyses. The results suggest that the stimuli that cause decreases in milk precede ionospheric perturbations. Therefore, the similarity between the lag times of the different precursors before the Tohoku earthquake might not indicate a direct causal relationship between milk yields and ionospheric perturbations but rather that triggers of decreased milk yields and ionospheric perturbations can occur simultaneously. Identifying stimuli that cause decreases in milk yield is important to help clarify the mechanism by which earthquake precursors lead to UAB. Anomalies in ultralow frequency (ULF, <10Hz) magnetic fields from the lithosphere are a candidate signal because the lag time between these anomalies and earthquakes is similar to that of decreased milk yields. Many studies have examined ULF radiation prior to earthquakes [7,8,47], and some studies reported the leading times were between a few weeks and a month [4,48]. Some reports suggest that the ULF magnetic field affects behavior and hormones [49,50,51,52,53]. The electromagnetic field with a frequency of 10 Hz is known to affect circadian activity rhythms [52,53]. Mahdavi et al. [49,51] have reported that exposures to electromagnetic fields with 5 Hz or 12 Hz elevated activity levels and adrenocorticotropic hormone concentrations in rats. Additionally, there have been reports that cattle aligned their body axes along geomagnetic field lines, which indicates they have the sense of magnetoreception [54,55,56]. To verify this hypothesis, observations of daily cow milk yields and ULF radiation from the same period in the same region need to be performed. There is an interesting report that discussed possible mechanisms for UAB prior to earthquakes based on observational data [22]. This study shows that the amount of wildlife (i.e., mammals and birds) captured by motion-triggered cameras in a national park decreased prior to the Contamana earthquake (M = 7.0) in the Peruvian Andes, and the lag time between these behavioral changes and the earthquake was coincident with the VLF propagation anomalies. They have suggested that air ionization due to positive hole carriers is a possible trigger for UAB and VLF propagation anomalies. Our results seem to conflict with their report. However, the observed behaviors and animal species differed between the two studies. We have shown the leading time of UAB differed depending on species [28]. Thus, it should be noted that “milk yield in dairy cows” could decrease due to ULF radiation.

Finally, we discuss the applicability of daily cow milk yields as an objectively observable UAB prior to earthquakes. Milk yields in each individual have been measured every day by many institutes in the animal husbandry industry to manage and improve productivity. As cows are reared in various places, at least in Japan, it is possible to elucidate relationships between milk yields and M, distance from epicenters or depth from hypocenters by more long term observations of milk yields in many regions. In this study, we were only able to evaluate the relevance to earthquakes in a limited region because we only had data on milk yields from one location. Further studies targeted at earthquakes in various regions should be conducted to confirm whether the results of this study are more generally applicable.

## 5. Conclusions

Our key finding is that daily cow milk yields decreased approximately three weeks before earthquakes. The probability that earthquakes with M > 5.5 that satisfy the Dobrovolsky radius condition occurred was highest 14 to 21 days after decreases in milk yields of greater than 1.5σ for more than two consecutive days (PG = 6.8; H = 85.7%). However, future studies that include more earthquakes in various regions and more detailed analyses need to be performed to confirm the reliability of these statistical estimates; this study only included a maximum of 32 earthquakes. All earthquakes that followed decreases in milk yields also followed VLF/LF propagation anomalies, one of the major precursory phenomena; however, the milk yields decreased approximately one week earlier than anomalies in VLF/LF propagation.

## Figures and Tables

**Table 1:** Estimated parameters in Equation (1) for individual lactation curves (Mean ± Standard deviation).

a	b	c
27.1 ± 15.9	0.183 ± 0.520	0.00644 ± 0.01981

**Table 2:** Estimated parameters in Equation (4) and the critical point in the temperature–humidity index (x0).

a	b	c	x0
−0.24	17.86	0.27	74.60

**Table 3:** The format of cross-tabulation tables consisting of the presence or absence of earthquakes and anomalies in milk yields; a represents the number of earthquakes occurring in alarm days, b represents the number of earthquakes occurring in no alarm days; c represents the number of alarm days without targeted earthquakes; d represents the number of no alarm days without targeted earthquakes.

Earthquake	Alarm		Total
	Yes	No	
Yes	a	b	a + b
No	c	d	c + d
Total	a + c	b + d	a + b + c + d

**Table 4:** The definitions in anomalies of milk yields and targeted earthquakes based on each criterion; M = magnitude; DRC = Dobrovolsky radius condition; **σ** = standard deviation.

	Anomalies in Milk Yields		Targeted Earthquakes	
	σ <	Duration (Days) ≥	M >	Distance from Epicenters <
Criterion 1	−1.5	1	5.0	DRC
Criterion 2	−1.5	1	5.5	DRC
Criterion 3	−1.5	1	6.0	DRC
Criterion 4	−1.5	2	5.0	DRC
Criterion 5	−1.5	2	5.5	DRC
Criterion 6	−1.5	2	6.0	DRC
Criterion 7	−1.5	1	5.0	DRC + 250 km
Criterion 8	−1.5	1	5.5	DRC + 250 km
Criterion 9	−1.5	1	6.0	DRC + 250 km
Criterion 10	−1.5	2	5.0	DRC + 250 km
Criterion 11	−1.5	2	5.5	DRC + 250 km
Criterion 12	−1.5	2	6.0	DRC + 250 km

**Table 5:** The cross-tabulation tables based on twelve criteria.

**Criterion 1**				**Criterion 7**			
**Earthquake**	**Alarm**		**Total**	**Earthquake**	**Alarm**		**Total**
	**Yes**	**No**			**Yes**	**No**	
Yes	7	3	10	Yes	11	21	32
No	103	231	334	No	66	246	312
Total	110	234	344	Total	77	267	344
**Criterion 2**				**Criterion 8**			
**Earthquake**	**Alarm**		**Total**	**Earthquake**	**Alarm**		**Total**
	**Yes**	**No**			**Yes**	**No**	
Yes	6	1	7	Yes	7	8	15
No	109	228	337	No	90	239	329
Total	115	229	344	Total	97	247	344
**Criterion 3**				**Criterion 9**			
**Earthquake**	**Alarm**		**Total**	**Earthquake**	**Alarm**		**Total**
	**Yes**	**No**			**Yes**	**No**	
Yes	3	1	4	Yes	4	2	6
No	124	216	340	No	116	222	338
Total	127	217	344	Total	120	224	344
**Criterion 4**				**Criterion 10**			
**Earthquake**	**Alarm**		**Total**	**Earthquake**	**Alarm**		**Total**
	**Yes**	**No**			**Yes**	**No**	
Yes	6	4	10	Yes	7	25	32
No	35	299	334	No	30	282	312
Total	41	303	344	Total	37	307	344
**Criterion 5**				**Criterion 11**			
**Earthquake**	**Alarm**		**Total**	**Earthquake**	**Alarm**		**Total**
	**Yes**	**No**			**Yes**	**No**	
Yes	6	1	7	Yes	6	9	15
No	37	300	337	No	33	296	329
Total	43	301	344	Total	39	305	344
**Criterion 6**				**Criterion 12**			
**Earthquake**	**Alarm**		**Total**	**Earthquake**	**Alarm**		**Total**
	**Yes**	**No**			**Yes**	**No**	
Yes	3	1	4	Yes	3	3	6
No	52	288	340	No	52	286	338
Total	55	289	344	Total	55	289	344

**Table 6:** The correspondence table of the anomalous milk yields, the observed targeted earthquakes and VLF data. Lat = Latitude, Lon = Longitude, D = Distance from epicenters, DRC = Dobrovolsky radius condition.

Anomalies of Milk Yields					Earthquake Data							VLF Data		
Start	End	Duration (Days)	Lead Time (Days)	σ (min)	Date (dd/mm/yyyy)	Lat	Lon	D (km)	DRC (km)	Depth (km)	M	Anomalies (Yes or No)	Lead Time (Days)	Path (Anomalous Day)
11/4/2014	12/4/2014	2	-	−3.57	-	-	-	-	-	-	-	-	-	-
16/04/2014	17/04/2014	2	18–19	−3.30	5/5/2014	34.95	139.48	132	380	156	6.0	Yes	13	JJY-KTU (22/04/2014)
31/05/2014	2/6/2014	3	14–16	−2.13	16/06/2014	36.62	141.80	165	282	37	5.7	Yes	10	JJY-KTU (06/06/2014)
					16/06/2014	37.07	141.16	149	312	52	5.8	Yes	10	JJY-NSB (06/06/2014)
23/06/2014	27/06/2014	5	15–19	−4.78	12/7/2014	37.05	142.32	228	1023	33	7.0	Yes	11	JJY-IMZ (01/07/2014)
29/07/2014	31/07/2014	3	-	−2.31	-	-	-	-	-	-	-	-	-	-
24/08/2014	26/08/2014	3	21–23	−1.90	16/09/2014	36.09	139.86	24	256	47	5.6	Yes	11	JJY-TYH (05/09/2014)
4/11/2014	5/11/2014	2	17–18	−2.93	22/11/2014	36.69	137.89	213	760	5	6.7	Yes	10	JJY-IMZ (12/11/2014)
-	-	-	-	-	20/12/2014	37.43	141.61	205	380	44	6.0	Yes	11	JJY-STU (09/12/2014)
8/12/2014	9/12/2014	2	16–17	−1.74	25/12/2014	37.23	141.65	191	256	36	5.6	Yes	8	JJY-NSB (17/12/2014)
